# Runs of Homozygosity Implicate Autozygosity as a Schizophrenia Risk Factor

**DOI:** 10.1371/journal.pgen.1002656

**Published:** 2012-04-12

**Authors:** Matthew C. Keller, Matthew A. Simonson, Stephan Ripke, Ben M. Neale, Pablo V. Gejman, Daniel P. Howrigan, Sang Hong Lee, Todd Lencz, Douglas F. Levinson, Patrick F. Sullivan, The Schizophrenia Psychiatric Genome-Wide Association Study Consortium

**Affiliations:** 1 Department of Psychology and Neuroscience, University of Colorado at Boulder, Boulder, Colorado, United States of America; 2 Institute for Behavioral Genetics, University of Colorado at Boulder, Boulder, Colorado, United States of America; 3 Center for Human Genetic Research, Massachusetts General Hospital, Boston, Massachusetts, United States of America; 4 Broad Institute of Harvard and MIT, Cambridge, Massachusetts, United States of America; 5 Analytic and Translational Genetics Unit, Massachusetts General Hospital, Boston, United States of America; 6 Department of Psychiatry and Behavioral Sciences, North Shore University Health System, Evanston, Illinois, United States of America; 7 Department of Psychiatry and Behavioral Sciences, University of Chicago, Chicago, Illinois, United States of America; 8 Queensland Institute of Medical Research, Brisbane, Australia; 9 Department of Psychiatry, Division of Research, The Zucker Hillside Hospital Division of the North Shore – Long Island Jewish Health System, Glen Oaks, New York, United States of America; 10 Center for Psychiatric Neuroscience, The Feinstein Institute for Medical Research, Manhasset, New York, United States of America; 11 Department of Psychiatry and Behavioral Science, Albert Einstein College of Medicine of Yeshiva University, Bronx, New York, United States of America; 12 Department of Psychiatry and Behavioral Sciences, Stanford University, Stanford, California, United States of America; 13 Department Department of Medical Epidemiology and Biostatistics, Karolinska Institute, Stockholm, Sweden; 14 Department of Genetics, The University of North Carolina at Chapel Hill, Chapel Hill, North Carolina, United States of America; 15 Department of Psychiatry, The University of North Carolina at Chapel Hill, Chapel Hill, North Carolina, United States of America; 16 Department of Epidemiology, The University of North Carolina at Chapel Hill, Chapel Hill, North Carolina, United States of America; Georgia Institute of Technology, United States of America

## Abstract

Autozygosity occurs when two chromosomal segments that are identical from a common ancestor are inherited from each parent. This occurs at high rates in the offspring of mates who are closely related (inbreeding), but also occurs at lower levels among the offspring of distantly related mates. Here, we use runs of homozygosity in genome-wide SNP data to estimate the proportion of the autosome that exists in autozygous tracts in 9,388 cases with schizophrenia and 12,456 controls. We estimate that the odds of schizophrenia increase by ∼17% for every 1% increase in genome-wide autozygosity. This association is not due to one or a few regions, but results from many autozygous segments spread throughout the genome, and is consistent with a role for multiple recessive or partially recessive alleles in the etiology of schizophrenia. Such a bias towards recessivity suggests that alleles that increase the risk of schizophrenia have been selected against over evolutionary time.

## Author Summary

Inbreeding occurs when genetic relatives have offspring. Because all humans are related to one another, even if very distantly, all people are inbred to various degrees. From a genetic standpoint, it is well known that inbreeding increases the risk that a child will have a rare recessive genetic disease, but there is also increasing interest in understanding whether inbreeding is a risk factor for more common, complex disorders such as schizophrenia. In this investigation, we used single-nucleotide polymorphism data to quantify the degree to which 9,388 schizophrenia cases and 12,456 controls were inbred, and we tested the hypothesis that people whose genome shows higher evidence of being inbred are at higher risk of having schizophrenia. We estimate that the odds of schizophrenia increase by ∼17% for every 1% increase in inbreeding. This finding is consistent with a role for multiple recessive or partially recessive alleles in the etiology of schizophrenia, and it suggests that genetic variants that increase the risk of schizophrenia have been selected against over evolutionary time.

## Introduction

Schizophrenia is a highly (.70–.80) heritable [1] neurodevelopmental disorder that has a lifetime prevalence of ∼0.4% [2]. As with most complex disorders, the specific genetic variants that account for a majority of the heritability of schizophrenia remain to be discovered. Two primary factors may explain the difficulty in identifying risk variants. First, the results of genome-wide association studies (GWAS) make it clear that a very large number of genes contribute to schizophrenia risk, and the overall population risk attributable to any one risk variant must be small [3]. Second, although common causal variants almost certainly play an important role in the genetic etiology of schizophrenia [4], [5], it is likely that the frequency distribution of schizophrenia risk alleles is biased towards the rare end of the spectrum [5]. Both of these factors are consistent with selection keeping schizophrenia risk alleles with the largest effects rare, such that no single allele can contribute much to population risk.

If schizophrenia risk alleles have been selected against across evolutionary time (have been under “purifying” selection), another prediction is that schizophrenia risk alleles will be biased towards being recessive. This bias, called directional dominance, occurs in traits subject to purifying selection because selection more efficiently purges the additive and dominant alleles with the strongest effects, leaving the remaining pool of segregating alleles more recessive than otherwise expected [6]. Directional dominance has traditionally been inferred from observations of inbreeding depression, the tendency for offspring of close genetic relatives to have higher rates of congenital disorders and lower fitness [7]. Fitness traits such as survival, reproduction, resistance to disease, and predator avoidance tend to show more inbreeding depression than traits under less intense selection [8]. Interestingly, there are numerous reports of inbreeding effects on human complex traits such as heart disease [9], hypertension [10], osteoporosis [11], cancer [12], and IQ [13], [14].

Studies that have investigated inbreeding effects on schizophrenia using pedigree data suggest that close (e.g., cousin-cousin) inbreeding is a risk factor [15], [16], [17], [18], [19], [20], although three studies have failed to find the predicted effect [21], [22], [23]. However, close inbreeding cannot be a major contributor to schizophrenia risk in industrialized countries given its rarity (<1% of marriages) [24] and the modest increase in the odds of schizophrenia among highly inbred offspring (∼2- to 5-fold) [15], [16], [17], [18], [19]. Nevertheless, inbreeding is a matter of degree; when distant relatives are considered, everyone is inbred to some degree. It is likely that the parents of the vast majority of people alive today share a common ancestor within ∼15 generations [25]. Although such “distant” inbreeding would be prohibitively difficult to detect from pedigrees, it can leave signals in the genome that are detectable using genome-wide single nucleotide polymorphism (SNP) data.

The inbreeding coefficient of an individual, *F*, is defined as the probability that two randomly chosen alleles at a homologous locus within an individual are *identical by descent* (IBD, identical because they are inherited from a common ancestor) [26]. Homozygosity arising from the inheritance of two IBD genomic segments is termed autozygosity. Most estimates of *F* assume that marker data are independent, and provide an aggregate measure of homozygosity at measured variants across the genome [27]. Recently, however, several investigators have used *runs of homozygosity* (ROHs; long stretches of homozygous SNPs) to infer autozygososity, and have investigated whether the proportion of the genome that exists in such ROHs, *Froh*, predicts complex traits [28], [29], [30], [31], [32], [33], [34], [35].

Of several alternative estimates of *F*, including *F* estimated by treating markers independently and *F* estimated from pedigree information, Keller, Visscher, and Goddard [25] recently concluded that *Froh* is optimal for inferring the degree of genome-wide autozygosity and for detecting inbreeding effects. However, given the small variation in genome-wide *Froh* in unselected samples (e.g., SD ∼.005), large sample sizes (e.g., >12,000) are necessary to detect inbreeding depression for likely effect sizes in samples not selected for recent inbreeding [25]. Studies investigating the effects of *Froh* on human complex traits with samples sizes <3,000 and that failed to find significant inbreeding effects [28], [33], [34], [35], [36] are likely to have been underpowered. That said, the only study of *Froh* in schizophrenia [29] found a very large inbreeding effect, but the effect was observed in a small sample (*n* = 322) and was significant only for ROHs caused by common haplotypes.

The present study uses imputed SNP data from 17 schizophrenia case-control datasets (total *N* = 21,844) that are part of the Psychiatric GWAS Consortium (PGC) [3], [37] to investigate whether *Froh* is associated with higher risk of schizophrenia. We also use an ROH mapping approach to investigate whether specific areas of the genome are predictive of case-control status when autozygous. This study represents the largest investigation to date on the potential consequences of autozygosity as estimated using *Froh*, and may help elucidate the genetic architecture and natural history of schizophrenia.

## Results

**Figure 1:**
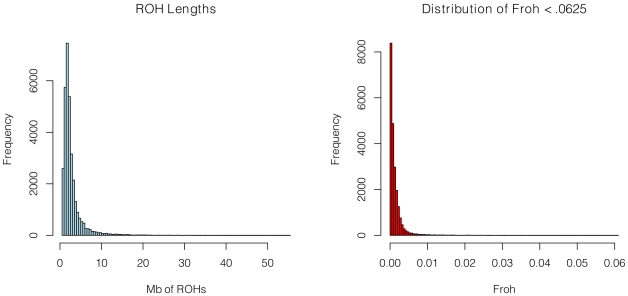
Distributions of ROH Lengths (left) and *Froh* (right) in the total sample. Distributions are based on ROHs from the imputed SNP data. For clarity, the distribution of *Froh* leaves omits 15 individuals who have *Froh*>.0625.

SNP data from 9,388 schizophrenia cases and 12,456 controls were collected with institutional review board approval from 17 sites in 11 countries (Table 1). Due to the different SNP platforms used across datasets, the number of SNPs remaining after quality control and linkage-disequilibrium pruning procedures (see below) differed substantially between the datasets (column 6 of Table 1). This induced artifactual differences in ROH statistics across datasets and made it impossible to allelically match ROHs across datasets (see Methods). To circumvent these issues, our main analysis concentrated on ROH results from a common set of imputed SNPs, but we also report results from the raw (non-imputed) SNP data. We imputed 1,252,901 autosomal SNPs in each dataset using BEAGLE [38] and HapMap3 as the reference panel [3]. We used extremely stringent imputation QC thresholds that have been shown to achieve accuracy rates similar to those in genotyped SNPs [39], leaving 398,325 high-quality imputed SNPs. We then removed 303,513 SNPs that were in high linkage disequilibrium (LD) with other SNPs. We defined ROHs as being ≥65 consecutive homozygous SNPs in a row (∼2.3 Mb) among the remaining 94,812 imputed SNPs [40]. We followed the same procedure for each dataset using the raw data, but defined ROHs as being ≥110 consecutive homozygous SNPs in a row (∼1.7 to ∼3.2 Mb, depending on the dataset). ROH thresholds were determined empirically (see *Methods*) so as to maximize the significance of the schizophrenia-*Froh* relationship, but as shown below, results differed little for alternative thresholds. *Froh* was defined as the proportion of an individual's genome that exists in ROHs. Descriptive statistics of ROHs and *Froh* across individual and combined datasets are shown in Table 1, and distribution of ROH lengths and *Froh* are shown in Figure 1 (Figure S1 shows the non-truncated distribution of *Froh*).

**Table 1:** Descriptive statistics of SNPs and ROHs (derived from imputed data) across datasets.

					LD pruned	% imputed	avg Froh	SD(Froh)	avg ROH	SD(ROH
Dataset	n	site	platform	SNPs passing QC	SNPs passing QC	SNPs genotyped	* 100	*100	length (kb)	length in kb)
AB	1418	UK	Affy 5	362461	180127	37%	0.13	0.21	2458	1897
BON	1778	Germany	Illum 550	494953	269497	21%	0.11	0.17	2424	2055
BULG	1135	Bulgaria	Affy 6	654278	288366	34%	0.21	0.45	3720	4014
CARWTC	3406	UK	Affy 500	365456	184440	37%	0.12	0.37	2563	2629
CAT2	606	USA	Affy 500	384538	234201	34%	0.18	1.05	3248	4107
DK	939	Denmark	Illum 650	533191	277949	20%	0.14	0.36	2544	2766
DUB	1130	Ireland	Affy 6	642723	271644	32%	0.16	0.21	2620	2237
EDI	650	UK	Affy 6	646310	275252	32%	0.11	0.21	2413	2272
MGS2	5163	USA/Australia	Affy 6	638937	280522	35%	0.11	0.21	2420	2021
MUC	785	Germany	Illum 317	295593	202543	20%	0.14	0.39	2901	3317
PORT	561	Portugal	Affy 5	333136	186277	39%	0.61	1.06	4662	4087
SW1	335	Sweden	Affy 5	330113	170520	35%	0.19	0.31	2844	2593
SW2	618	Sweden	Affy 6	661602	275690	31%	0.23	0.37	2911	2782
TOP3	598	Norway	Affy 6	630195	280545	32%	0.15	0.17	2375	1575
UCLA	1334	Netherlands	Illum 550	505922	270851	21%	0.15	0.37	2728	2681
UCL	1009	UK	Affy 5	277652	156506	36%	0.09	0.13	2212	1633
ZHH	379	USA	Affy 500	258470	159220	37%	0.36	0.99	3551	3385
Total	21844	N/A	Imputed	398325	94812	82%†	0.15	0.40	2788	2771

**†:** 82% of imputed SNPs used in overall ROH analysis were genotyped on at least one platform.

### ROH burden results

**Figure 2:**
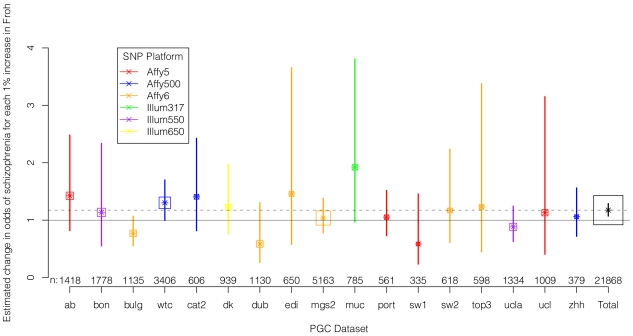
Estimated changes in odds of schizophrenia for each 1% increase in *Froh* (odds ratios; asterisks) and their 95% confidence intervals (bars) across the 17 datasets (colored) and for the total sample (black) from the imputed SNP data. Boxes are proportional to the square root of sample sizes (also shown at the bottom). Dataset names are on the x-axis. Although none of the estimated odds ratios are significantly different from one individually, the overall effect (black) is highly significant.

We regressed case-control status on *Froh* separately in each of the 17 datasets using logistic regression, controlling for potential confounding factors such as population stratification and SNP quality metrics (see *Methods*). Figure 2 shows the estimated change in odds of schizophrenia for every 1% increase in *Froh* and the 95% confidence intervals from these 17 logistic regression equations, and Figure S2 shows the same results from an analysis conducted on the raw (non-imputed) SNP data. It should be noted that confidence intervals are symmetric on the log odds scale but asymmetric on the odds ratio scale shown in Figure 2 and Figure S2. As indicated by the confidence intervals, there was a great deal of variability in the estimates of the *Froh*-schizophrenia association, and none of these 17 odds ratios significantly differed from one. Nevertheless, 13 of the odds ratios were greater than one (i.e., consistent with autozygosity being a schizophrenia risk factor) while 4 were less than one, a result inconsistent with chance (exact binomial test, *p* = 0.025). More formally, using a mixed linear effects logistic regression model that treated dataset as a random factor (which also controlled for SNP platform because dataset was nested within each platform), the overall association between schizophrenia and *Froh* in the combined sample was highly significant (β = 16.1, *z* = 3.44, *p* = 0.0006 in the imputed data, and β = 17.98, *z* = 3.89, *p* = 0.0001 in the raw data). A slope of *Froh* on schizophrenia of 16.1 is interpreted as saying that for every 0.01 increase in *Froh*, the odds of schizophrenia are multiplied by 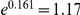, or increased by 17%.

**Figure 3:**
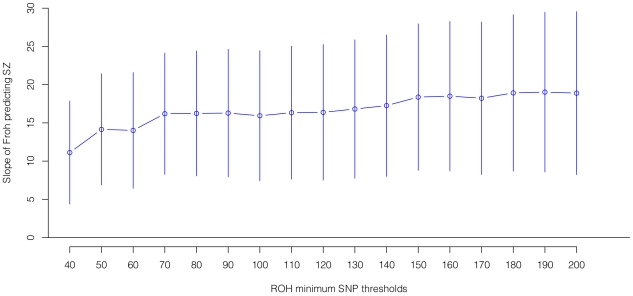
Slope estimates (the change in log odds for a 1% increase in *Froh*; points) and their 95% confidence intervals (bars) of *Froh* from imputed SNP data predicting schizophrenia for different SNP homozygosity thresholds of calling ROHs. Minimum SNP thresholds for full and reduced models are offset for clarity. All ROH thresholds were significant; the most significant result was for ROHs defined as being 65 or more homozygous SNPs in a row.

Several secondary analyses were undertaken to explore the robustness and generality of the *Froh*-schizophrenia association. There was no evidence that the *Froh*-schizophrenia association differed significantly between datasets (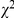 = 0.253, *p* = 0.88), and the association remained highly significant in 17 models that removed one dataset at a time. To understand if this association was sensitive to the covariates included in the model, we ran additional models that controlled for no covariates, various combinations of covariates, and dataset-by-covariate interactions. In all of these models, the association between *Froh* and schizophrenia remained significant. We also found that our conclusions were insensitive to the SNP threshold used to define ROHs; the association between *Froh* and schizophrenia remained relatively unchanged and significant for all SNP thresholds of ≥40 consecutive SNPs in both the imputed (Figure 3) and raw (Figure S3) data. Finally, both common ROHs (β = 28.5, *z* = 2.51, *p* = .012), which arose from haplotypes that were observed often in the data, and uncommon ROHs (β = 20.4, *z* = 3.29, *p* = .001) were predictive of case-control status (see *Methods*).

### Autozygosity versus hemizygosity

Copy number variant deletions can create apparent ROHs in SNP data. We could not systematically catalog the overlap between deletions and ROHs in the full dataset because deletion information is not available on the entire sample. However, Levinson and colleagues [41] identified 501,890 deletions (using their “broad” criteria) in the MGS2 dataset (*n* = 5,163), comprising about one-fourth of the total sample used here. The median length of a deletion in the MGS2 dataset was ∼10 kb, whereas the median length of a ROH was ∼2,000 kb, suggesting that very few deletions would be long enough to qualify as ROHs. Consistent with this expectation, we found that only 10 of 6,480 ROHs in the MGS2 dataset were possible deletions using the algorithm described by McQuillan et al. [31], which called a ROH a “possible deletion” if its total length was <500 kb after removing deletion regions from ROHs. The percentage of ROHs thus classified (0.15%) was similar to the percentage (0.30%) reported by McQuillan et al. [31]. This percentage is too small to have a meaningful impact on our results, because when we removed a larger percentage of ROHs that were identified as being the largest schizophrenia risk factors (see below), the *Froh*-schizophrenia association remained highly significant. We conclude that ROH results reported above are due to autozygosity rather than hemizygosity.

### The effects of close versus distant inbreeding on schizophrenia

A reverse-causation explanation of the *Froh*-schizophrenia association is possible: people who have a higher “load” of schizophrenia risk alleles (and who transmit this risk to offspring) may be more likely to mate with a relative. This counter-explanation to the causal interpretation of the *Froh*-schizophrenia relationship is less likely if the relationship holds not only for close inbreeding, but also for autozygosity caused by distant and almost certainly unintended inbreeding (arising from common ancestors who lived many generations ago). One way to investigate this issue is to remove positive outliers on *Froh* and reassess the *Froh*-schizophrenia relationship. We reran models after dropping a) two individuals with *Froh*>0.125, the approximate equivalent of half-sibling inbreeding (β = 15.57, 95% CI(β) = [25.0, 6.14], *z* = 3.24, *p* = 0.001); b) 15 individuals with *Froh*>0.0625, the approximate equivalent of cousin-cousin inbreeding (β = 15.13, 95% CI(β) = [26.1, 4.25], *z* = 2.73, *p* = 0.006); c) 56 individuals with *Froh*>0.03125, the approximate equivalent of half-cousin inbreeding (β = 8.43, 95% CI(β) = [21.43, −4.55], *z* = 1.27, *p* = 0.20); d) 942 individuals with *Froh*>.005, consistent with elevated levels of distant inbreeding (β = 5.17, 95% CI(β) = [34.84, −24.50], *z* = 0.34, *p* = .73); and e) 6,101 individuals with *Froh* scores above the mean level of *Froh* (β = 66.91, 95% CI(β) = [139.2, −5.4], *z* = 1.81, *p* = .07). To test whether the change in significance after dropping outliers was due to the *Froh*-schizophrenia association being stronger for individuals with high levels of autozygosity, we included a quadratic term (*Froh^2^*) in the regression model. In contrast to the highly significant linear term of *Froh*, the quadratic term of *Froh* was non-significant (*p* = .09), suggesting that the effect of autozygosity is linear across the range of *Froh* observed here.

**Figure 4:**
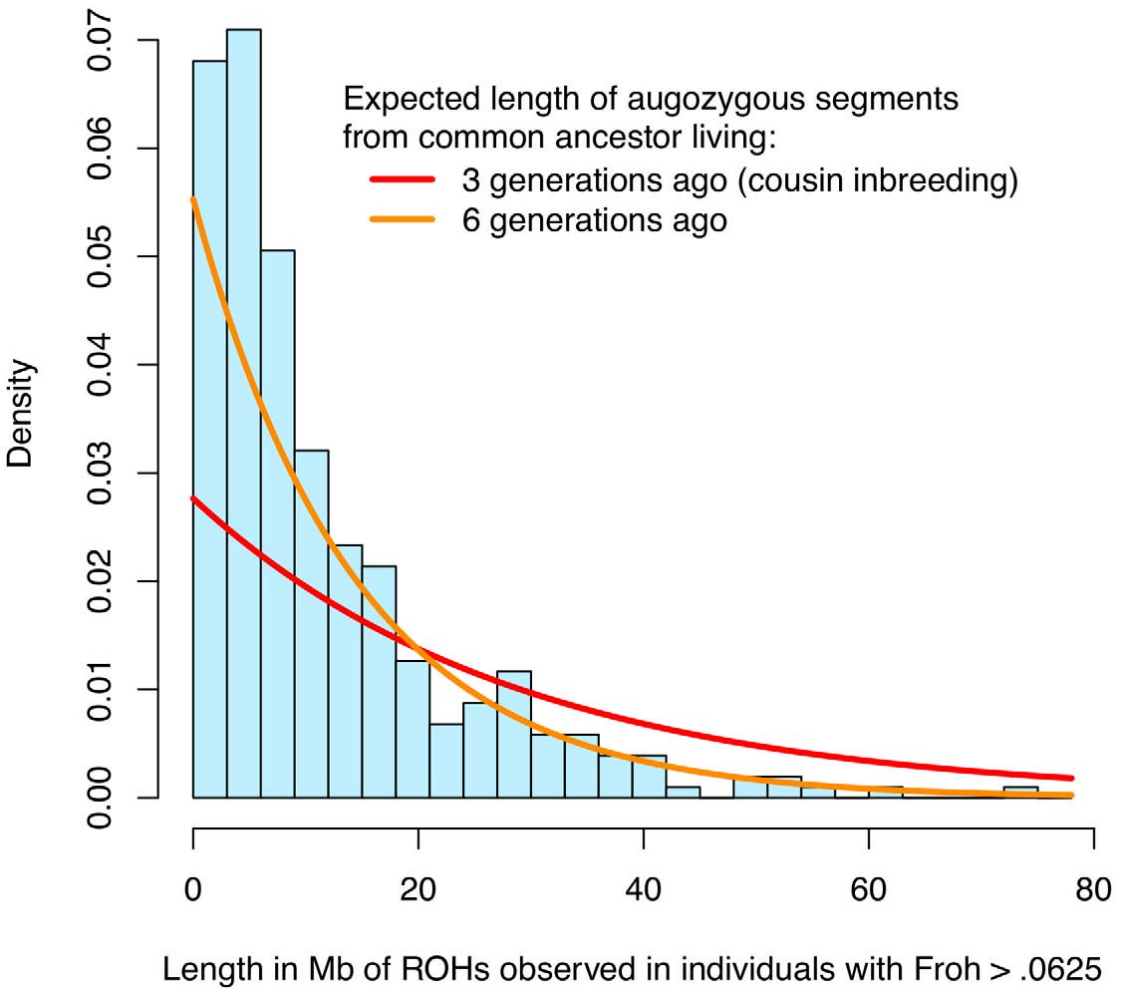
Distribution of ROH lengths for the 15 individuals with *Froh*>.0625 in the sample (blue) and the expected lengths of autozygous segments for different levels of inbreeding (red and orange). Nearby ROHs that were broken up by a possible heterozygous SNP miscall were joined together. Assuming Haldane's recombination model, the length of an autozygous segment should follow an exponential distribution with mean equal to 1/(2×number of generations since the common ancestor) in Morgans. The figure shows that the distribution of ROH lengths among individuals with *Froh*>.0625 is most consistent with autozygosity caused by common ancestors between parents who lived ∼6 generations ago.

The simple approach—dropping outliers—to distinguishing the effects of distant versus close inbreeding is problematic for two reasons. First, *Froh* is naturally extremely right-skewed (Figure 1 and Figure S1), even in large, simulated populations where close inbreeding is disallowed [25], and so dropping even a small number of outliers greatly reduces the variation in *Froh*, decreases the statistical power to detect an association, and degrades the precision of point estimates. Indeed, there is no evidence that the schizophrenia-*Froh* association changes as outliers are removed, because the original point estimate (β = 16.1) is contained within every confidence interval above. Thus, the results from dropping outliers demonstrate that the *Froh*-schizophrenia relationship is not driven by a few highly inbred individuals, but do not allow us to distinguish the effects of distant vs. close inbreeding. Second, individuals with high *Froh* can arise by chance from the accumulation of many paths of distant inbreeding [25], and are not necessarily the products of close inbreeding. For example, the distribution of lengths of observed ROHs among individuals with *Froh*>0.0625 is more consistent with inbreeding from common ancestors living ∼6 generations ago than with first cousin inbreeding (Figure 4).

An alternative and more robust approach for assessing the relative importance of distant versus close inbreeding is to compare the effects of short versus long ROHs. We defined *Froh<5 Mb* as the proportion of the autosome in ROHs of length <5 Mb and *Froh>5 Mb* as the converse, with 5 Mb chosen as the threshold because the variances of *Froh<5 Mb* and *Froh>5 Mb* were equal. An autozygous segment spanning <5 Mb should originate from a common ancestor ≥10 generations ago on average [41]. The effect of *Froh<5 Mb* (β = 27.6, *z* = 2.23, *p* = 0.026) was similar to the effect of *Froh>5 Mb* (β = 24.3, *z* = 2.01, *p* = 0.044), consistent with the hypothesis that autozygosity arising from distant inbreeding is about as much of a schizophrenia risk factor as autozygosity arising from more recent common ancestors.

### ROH mapping analysis

**Figure 5:**
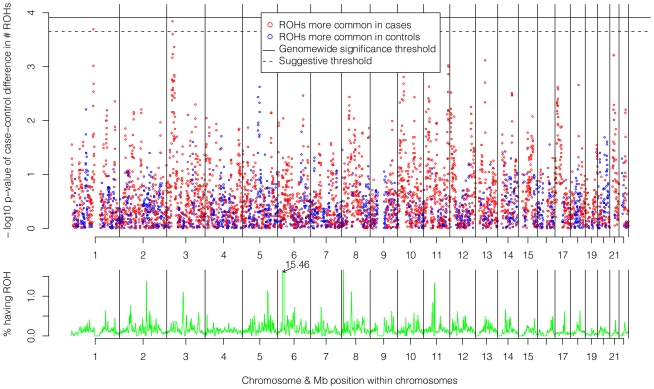
Risk and protective effects of ROHs on schizophrenia risk and frequencies of ROHs across the autosome. Top panel: −log_10_ p-values for the risk (red) and protective (blue) effects of ROHs on schizophrenia risk at each 500 kb region along the autosome. Bottom panel: frequencies of ROHs across the autosome.

The top of Figure 5 shows the −log_10_ p-values for the 5,742 logistic regressions predicting case-control status from ROHs at each 500 kb bin along the autosome. No regions reached genome-wide significance although two (1p13.2 and 3p24.1) exceeded the “suggestive significance” threshold. Table 2 shows the twelve genes located in these two regions along with their potential functional significances. Neither region has been previously implicated in linkage analyses [42], copy number variant analyses [43], or GWAS meta-analyses [3] of schizophrenia. After recalculating *Froh* with the two suggestively significant regions removed, results of the burden analysis remained essentially unchanged, showing that these regions have only a minor influence on the overall *Froh*-schizophrenia association and suggesting that the effect of autozygosity is diffused across the genome.

**Table 2:** Genes within the two 500 kb bins that reached “suggestive” significance in the ROH mapping analysis.

Gene	Location	Functional Significance
MRP63P1	1p13.2	mitochondrial ribosomal protein 63 pseudogene
LOC100421116	1p13.2	involved with trafficking protein, a kinesin binding 2 pseudogene
TRIM33	1p13.2	thought to be a transcriptional co-repressor
RPL26P10	1p13.2	ribosomal protein pseudogene
EIF2S2P5	1p13.2	subunit 2 beta pseudogene
LOC643586	1p13.2	pyruvate kinase, muscle pseudogene
BCAS2	1p13.2	believed to play a role in increasing estrogen receptor function
DENND2C	1p13.2	gene of unknown function; highly conserved across mammals
AMPD1	1p13.2	catalyzes deamination of AMP to IMP in skeletal muscle; role in the purine nucleotide cycle
RBMS3	3p24.1	encodes an RNA-binding protein; implicated in smoking cessation, diabetes, and bone mass
RPS12P5	3p24.1	pseudogene of unknown function
LOC100129900	3p24.1	pseudogene of unknown function

The bottom of Figure 5 shows the frequencies of ROHs occurring at each 500 kb bin across the autosome. With one exception, less than 1.5% of the sample had an ROH at each region. The exception occurs in the Major Histocompatibility Complex region in 6p21.3, where 15.5% of the sample had an ROH. This high number of ROHs is explained by the low recombination and long, common, geographically-specific haplotypes that occur here [44], [45].

## Discussion

These results suggest that the odds of schizophrenia increase by ∼17% for every 0.01 increase in the proportion of estimated autosomal autozygosity (*Froh*). Given the standard deviation of *Froh* (0.004), this effect is modest, explaining <0.1% of the risk of schizophrenia in outbred populations (Nagelkerke *r* = 0.026). Nevertheless, this effect implies that close inbreeding is a significant risk factor for schizophrenia. Cousin-cousin inbreeding is predicted to increase the odds of schizophrenia 2.74-fold (by 174%) and second-cousin inbreeding is expected to increase the odds of schizophrenia 1.29-fold (by 29%). These estimates are roughly in line with previous reports on schizophrenia from samples selected based on pedigree inbreeding [15], [16], [17], [18], [19], [20] and similar to the increased risk of major birth defects following close inbreeding [46]. Given that second cousin or closer inbreeding occurs frequently in several world cultures, and that progeny from such unions account for about 10% of the world's population [47], autozygosity may be an important risk factor for schizophrenia worldwide.

The apparent effect of autozygosity on schizophrenia suggests that risk alleles that are more dominant have disappeared over evolutionary time at a faster rate than risk alleles that are more recessive. This is consistent with the hypothesis that alleles that increase the risk of schizophrenia have been under purifying (negative) selection [48].

There are three main limitations to the current study. The most important is that this was a mega-analysis of SNP data collected at 17 different sites using six different platforms. The collection and handling of samples, the distribution of samples on plates, and the calling of SNPs differed between and within sites in ways that were impossible to quantify in the analysis. This certainly added noise to the results, reducing the apparent effect size, but also may have introduced subtle biases. We have tried to statistically control for as many of these as possible, but the possibility remains that uncorrected biases made these results appear stronger or weaker than they actually are.

Second, while our results clearly support the hypothesis that autozygosity is a risk factor for schizophrenia, they are less clear about how confidently we can differentiate the roles of distant versus close inbreeding. On one hand, when enough outliers on *Froh* values are excluded, the case-control difference is no longer significant. On the other hand, there are good statistical reasons to consider the analysis of short versus long ROHs more valid than the analyses that exclude individuals with the highest *Froh* values. Thus, the authors favor the conclusion that both distant and close inbreeding are risk factors for schizophrenia. A more definitive answer to this question would either require a substantially larger sample size or a sample of similar size to the current one but drawn from a population with greater variation in levels of distant inbreeding.

A final limitation has to do with the correlational nature of these findings. We argue that the *Froh*-schizophrenia association is likely to be causal because the association is consistent with a known genetic mechanism, directional dominance, and because the association appears to be as robust for short ROHs as long ROHs. Short ROHs are likely to represent autozygosity caused by distant inbreeding, and therefore seem less likely to differ between parents as a function of their load of schizophrenia risk alleles. Nevertheless, we cannot eliminate the possibility that parents of offspring who have schizophrenia differ in ways that make distant inbreeding more likely, such as an increased propensity to mate with individuals who have culturally, geographically, or ethnically similar backgrounds.

### Conclusion

Inbreeding has had a central place in population genetics since its inception, but until recently, the effects of inbreeding could only be investigated from careful analysis of pedigrees and only for close inbreeding. SNP data allows investigation into the effects of potentially very distant inbreeding in non-selected samples, and allows insight into where the signal comes from in the genome. However, unless samples are specifically selected based on inbreeding, very large samples are required to reliably detect effects of autozygosity due to the low variation between individuals in their levels of autozygosity. The present investigation used SNP data from a large sample to conclude that autozygosity is a risk factor for schizophrenia. If the relationship between *Froh* and schizophrenia is due to directional dominance, such that schizophrenia risk alleles are more recessive than otherwise expected, this suggests that alleles that increase the risk of schizophrenia have been under negative selection ancestrally.

## Methods

### Psychiatric GWAS consortium data

Full methods are given elsewhere [3]. Briefly, 9,388 schizophrenia cases and 12,456 controls were collected with institutional review board approval from 17 sites in 11 countries (Australia, Bulgaria, Denmark, Germany, Ireland, Netherlands, Norway, Portugal, Sweden, United Kingdom, and Unites States of America; see Table 1). As is typical in the field, individuals with schizophrenia or schizoaffective disorder were included as cases [49] [50]. The quality of phenotypic data was verified by a systematic review of data collection methods to ensure consistency between sites.

### Quality control (QC) procedures for raw SNP data

The initial set of samples and SNPs passed common GWAS QC procedures [3]. In particular, we removed a) one individual from any pair of individuals who were related with pi-hat >0.2, b) individuals with non-European ancestry as determined by principal components analysis; c) samples with SNP missingness >0.02; or d) samples with genome-wide heterozygosities >6 standard deviations above the mean. SNPs were excluded if they a) deviated from Hardy-Weinberg equilibrium at p<1×10^−6^; b) had missingness >0.02; c) showed a minor allele frequency difference to HapMap CEU>0.15; or d) had a missingness difference between cases and controls >0.02. On average the QC processes excluded 15 individuals (0–100) and 38K SNPs (5K–160K) per dataset. The number of SNPs per dataset after QC varied between 250K and 680K (Table 1).

### Imputation and QC procedures for imputed SNP data

Six different SNP platforms (Affymetrix 500K, 5.0, and 6.0 chips along with the Illumina 317K, 550K, and 650K chips; Table 1) were used across the 17 datasets. Differences across platforms in SNP densities, frequency distributions, LD patterns, and missingness led to variation in ROH statistics across datasets. For example, the DK dataset contains 280K SNPs after LD pruning (1 SNP per 11 kb) whereas the UCL datset contains 156K SNPs after LD pruning (1 SNP per 21 kb). ROHs therefore would have to be about twice as long in the UCL dataset to qualify, which induces artifactual noise in ROH statistics due to platform effects. This issue is not circumvented by using an ROH threshold based on length rather than number of SNPs; in this case, half as many homozygous SNPs in a row would be required to call an ROH in the less dense dataset. In both cases, the type-I and type-II error rates of autozygosity detection differ systematically between datasets.

To overcome these issues, we imputed dosages for 1,252,901 autosomal SNPs in each dataset using BEAGLE [38] and HapMap3 as the reference panel [3]. We converted imputation dosages to best-guess (highest posterior probability) SNP calls because ROH detection algorithms require discrete SNP calls. Because typical imputation QC thresholds can lead to a high number of missed ROHs, we used extremely stringent imputation QC thresholds that have been shown to achieve accuracy rates similar to those in genotyped SNPs [39]. In particular, we removed 854,566 imputed SNPs with dosage r^2^<0.90 in any dataset (the dosage r^2^ is equivalent to *MACH's* r^2^ measure described in [51]), that had a dosage r^2^<0.98 or >1.02 in the overall sample, or that had MAF<0.05, leaving 398,325 high-quality imputed SNPs. Because only ∼100K SNPs are use to make ROH calls (see below), we could afford to lose a large number of imputed SNPs from QC procedures.

ROHs called from imputed data were less variable across platform and across datasets in terms of basic descriptive statistics, in the effects of potential artifacts (e.g., SNP missingness rates and excess heterozygosity on *Froh*), and in their associations with schizophrenia. We therefore report results on ROHs called from imputed data. However, results for the ROHs called from raw data were similar, and are shown in Figures S2 and S3.

### ROH calling procedures

Of three programs investigated (PLINK, GERMLINE, and BEAGLE), a recent investigation by three of the authors of the current report [40] concluded that PLINK (using the –homozyg commands) optimally detected autozygous stretches and maximized power to detect an effect of autozygosity on a phenotype. In particular, the authors recommended: a) pruning for strong LD (removing any SNPs having a multiple R^2^>0.90 with all other SNPs in a 50 SNP window), which reduced false autozygosity calls by removing redundant markers in SNP-dense regions and by making SNP coverage more uniform; and b) defining ROHs as being ≥65 consecutive homozygous SNPs with no heterozygote calls allowed [40]. We used these recommendations to detect ROHs in all analyses, although to ensure that we did not miss potential effects of autozygosity, we report on results from the specific ROH threshold (number of homozygous SNPs in a row) that minimized the p-value of the *Froh*-schizophrenia association (see Figure 3 and Figure S3). This threshold was 65 SNPs-in-a-row (spanning ∼2.3 Mb) in the imputed SNP data and 110 SNPs-in-a-row (spanning ∼1.7 Mb to ∼3.2 Mb depending on the dataset) in the raw data. It should be noted that results were relatively insensitive to the specific threshold chosen (Figure 3 and Figure S3). Finally, to ensure that no ROH crossed a region of low SNP density (e.g., a centromere), we also required that ROHs have a density greater than 1 SNP per 200 kb, and we broke an ROH in two if a gap >500 kb existed between adjacent homozygous SNPs.

ROHs can also be categorized by their frequency (how often a particular haplotype creates ROHs at a given location). We used PLINK's –homozyg-group and –homozyg-match arguments to understand whether uncommon ROHs or common ROHs were particularly predictive of case-control status, defining ROHs in a given region as “uncommon” when they allelically matched with 16 (the median) or fewer other ROHs in the combined data; all other ROHs were defined as “common.”

### ROH burden analysis

For each individual, we summed the total length of all their ROHs in the autosome and divided by the total SNP-mappable autosomal distance (2.77×10^9^ bases) to derive *Froh*, the proportion (0 to 1) of the autosome in ROHs. *Froh* was used as the predictor of case-control status in ROH burden analyses. *Froh* can be influenced by confounding factors like population stratification (e.g., if background levels of heterozygosity or autozygosity differed by ancestry), low quality DNA leading to incorrect SNP calls, and heterozygosity levels that vary across plates, DNA sources, etc. To control for the effects of stratification, we included the first 20 principal components based on ∼30K SNPs genotyped in all datasets. We also controlled for the percentage of missing calls in the raw SNP data and excess heterozygosity as these track the quality of SNP calls [52]. Using simulations, Keller et al. [25] showed that the ability of *Froh* to accurately estimate autozygosity is negligibly affected by statistically controlling for excess heterozygosity, and therefore doing so should have minimal effect on results when genotyping error rates are low, but may help elucidate effects of ROHs when such errors are present.

We regressed case-control status on *Froh* separately in each of the 17 datasets using logistic regression, controlling for the potential confounders discussed above. We then employed a mixed linear effects logistic regression model (using the lme4 package in R version 2.11) to estimate the overall effect of *Froh* across datasets, treating dataset as a random factor. This also controlled for SNP platform because dataset was nested within each platform (controlling for platform was statistically redundant in a model also controlling for dataset).

### ROH mapping analysis

To understand whether any genomic area was predictive of case-control status, we divided the autosome into 5,742 segments of length 500 kb each. At each segment, an individual was scored as either having a ROH that partially or completely overlapped the segment or not. We performed 5,742 logistic regressions, regressing case-control status on whether or not individuals had an ROH in each segment, controlling for covariates described above. To derive a genome-wide significance threshold corrected for multiple testing, we permuted case-control status within the 17 datasets and reran the 5,742 logistic regressions, preserving the most significant result of each permutation. We repeated this permutation 1,000 times. The 50^th^ most significant p-value was the genome-wide significance threshold and the 100^th^ most significant p-value was the “suggestive” genome-wide significance threshold.

## Supporting Information

Figure S1 Distributions of ROH Lengths (left) and *Froh* (right) in the total sample, including individuals with *Froh*>.0625. Distributions are based on ROHs from the imputed SNP data.
(TIF)

Figure S2 Estimated changes in odds of schizophrenia for each 1% increase in *Froh* (odds ratios; asterisks) and their 95% confidence intervals (bars) across the 17 datasets (colored) and for the total sample (black) from the raw SNP data. Boxes are proportional to the square root of sample sizes (also shown at the bottom). Dataset names are on the x-axis. Although none of the estimated odds ratios are significantly different from one individually, the overall effect (black) is highly significant.
(TIF)

Figure S3 Slope estimates (the change in log odds for a 1% increase in *Froh*; points) and their 95% confidence intervals (bars) of *Froh* from raw SNP data predicting schizophrenia for different SNP homozygosity thresholds of calling ROHs. Minimum SNP thresholds for full and reduced models are offset for clarity. All ROH thresholds were significant; the most significant result was for ROHs defined as being 110 or more homozygous SNPs in a row.
(TIF)
