# Supplementary figures and images for: Runs of Homozygosity Implicate Autozygosity as a Schizophrenia Risk Factor
Source: PLoS Genet. 2012 Apr 12;8(4):e1002656. doi: 10.1371/journal.pgen.1002656 (PMC3325203; doi:10.1371/journal.pgen.1002656)

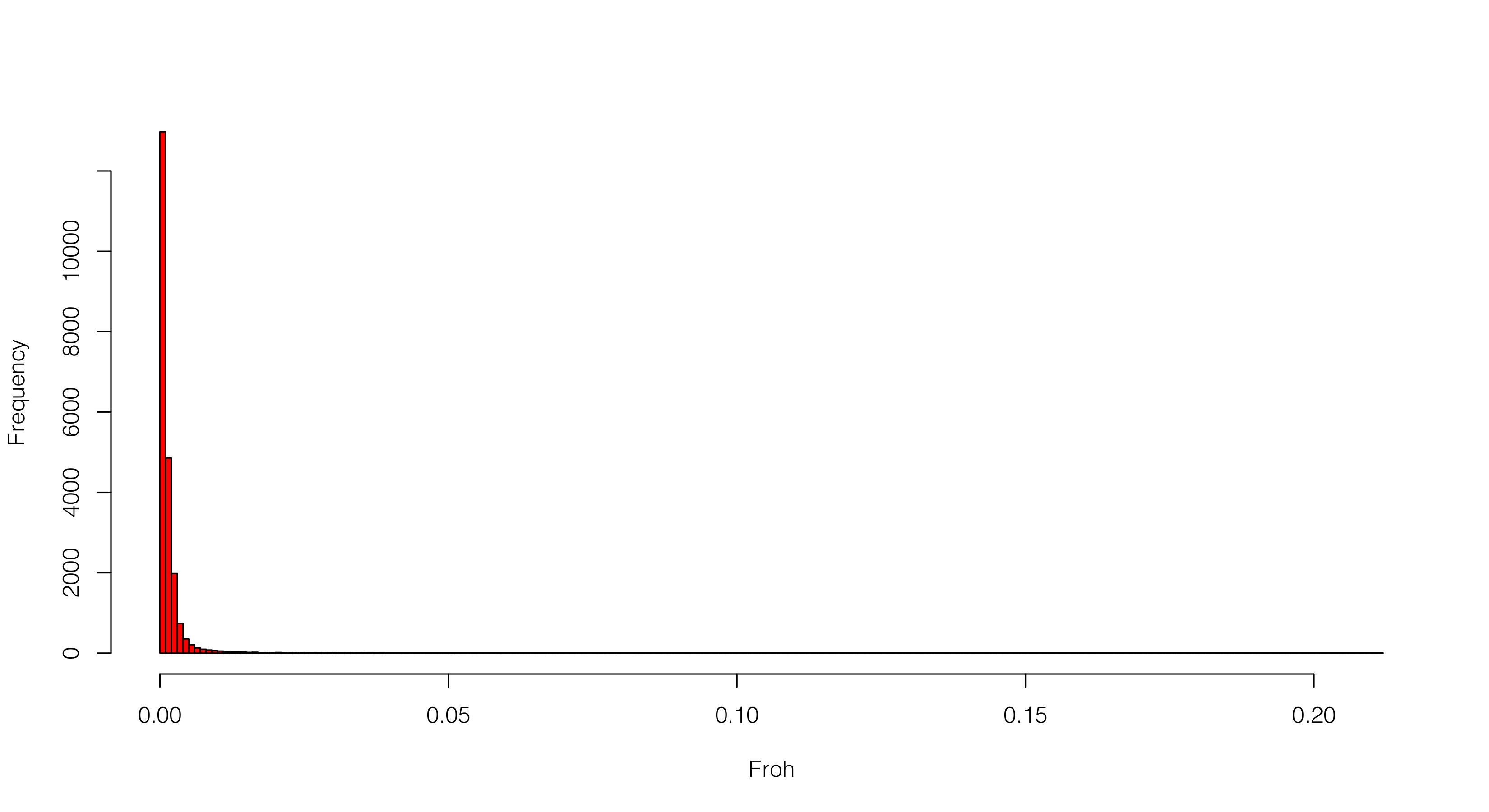

Supplement: Figure S1 — Distributions of ROH Lengths (left) and Froh (right) in the total sample, including individuals with Froh>.0625. Distributions are based on ROHs from the imputed SNP data. (TIF) [file pgen.1002656.s001.tif]

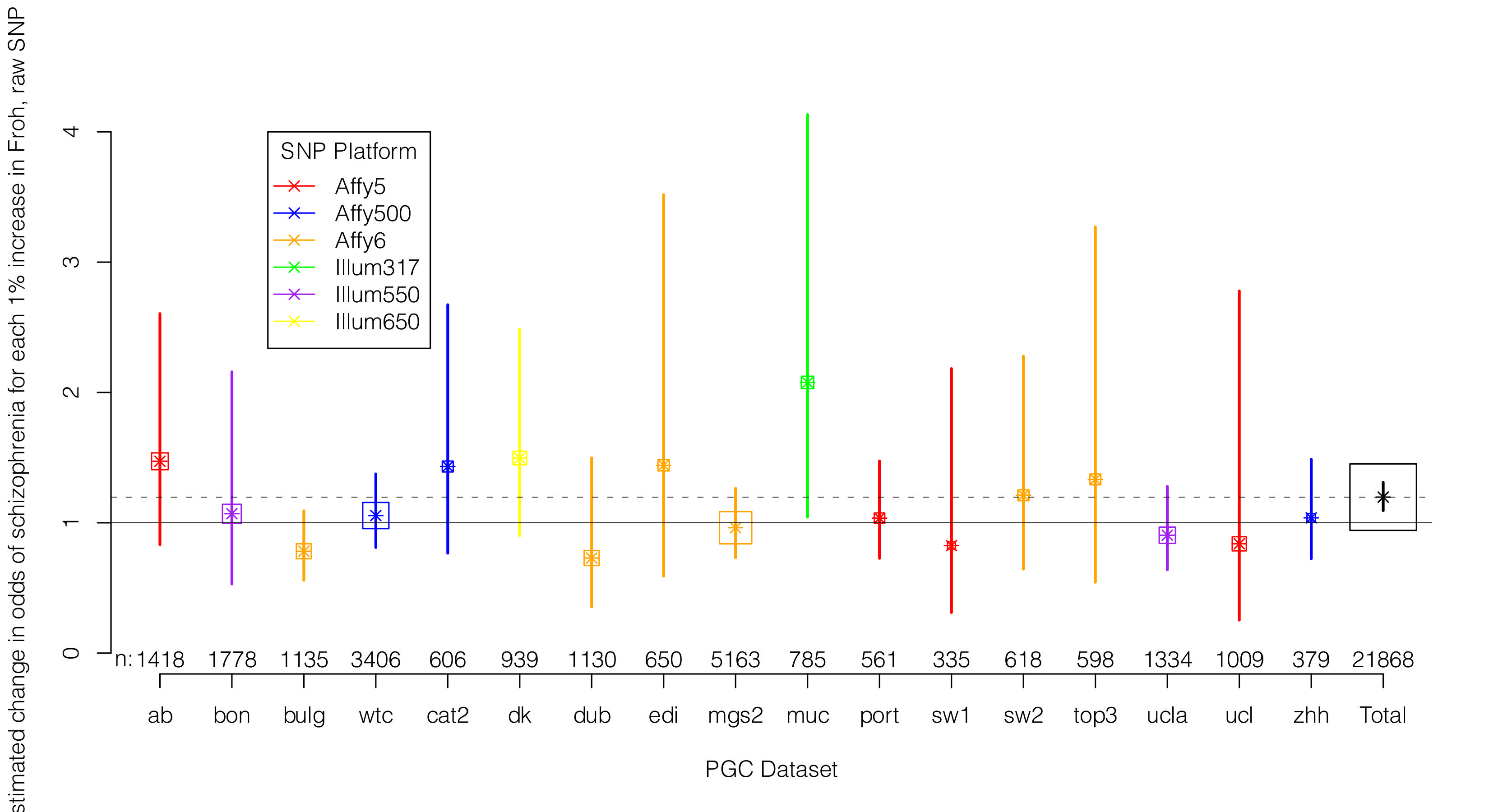

Supplement: Figure S2 — Estimated changes in odds of schizophrenia for each 1% increase in Froh (odds ratios; asterisks) and their 95% confidence intervals (bars) across the 17 datasets (colored) and for the total sample (black) from the raw SNP data. Boxes are proportional to the square root of sample sizes (also shown at the bottom). Dataset names are on the x-axis. Although none of the estimated odds ratios are significantly different from one individually, the overall effect (black) is highly significant. (TIF) [file pgen.1002656.s002.tif]

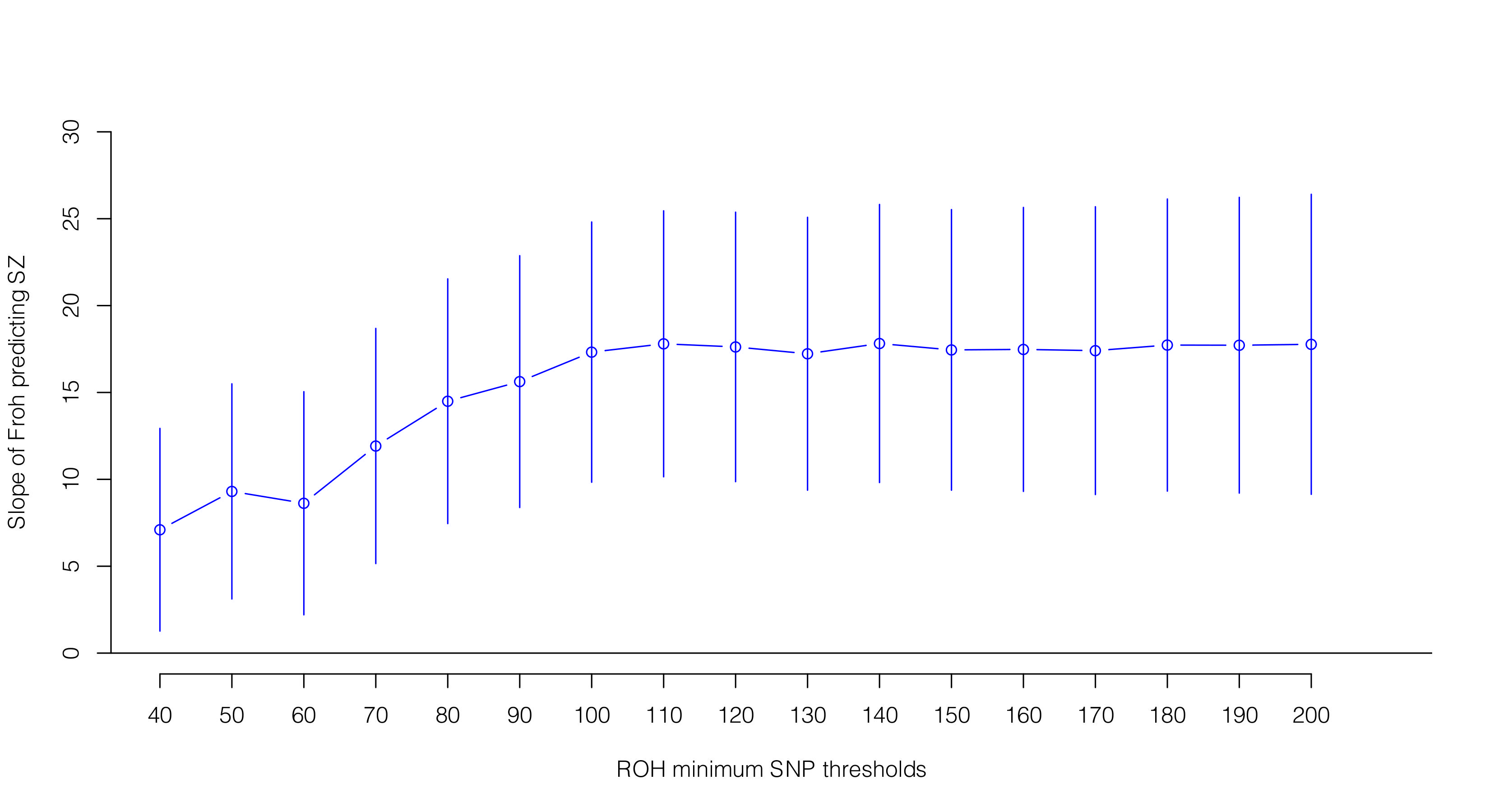

Supplement: Figure S3 — Slope estimates (the change in log odds for a 1% increase in Froh; points) and their 95% confidence intervals (bars) of Froh from raw SNP data predicting schizophrenia for different SNP homozygosity thresholds of calling ROHs. Minimum SNP thresholds for full and reduced models are offset for clarity. All ROH thresholds were significant; the most significant result was for ROHs defined as being 110 or more homozygous SNPs in a row. (TIF) [file pgen.1002656.s003.tif]
